# The relationship between parental perceptions of transition practices, school readiness and mental health in Chinese children: the moderating effects of sociodemographic factors

**DOI:** 10.3389/fped.2026.1677739

**Published:** 2026-08-25

**Authors:** Catalina Sau Man Ng, Victor C. W. Chan, Olivier Marchal

**Affiliations:** 1 Department of Early Childhood Education, Education University of Hong Kong, Hong Kong SAR, China; 2 Department of Business Administration, Gies College of Business, University of Illinois Urbana-Champaign, Urbana-Champaign, IL, United States

**Keywords:** Chinese children, mental health, moderation, school readiness, sociodemographic factors, transition practices

## Abstract

**Objective:**

Transitioning from kindergarten to primary school (K–P) represents a pivotal milestone in children's development. This study examined relationships among parental perceptions of transition practices, children's school readiness, and mental health outcomes, while exploring the moderating role of sociodemographic factors.

**Methods:**

Cross-sectional data were collected from 259 Chinese parents through a two-stage sampling process. In the first stage, primary schools were stratified by region (Hong Kong Island, Kowloon, and the New Territories), and a random sample of 21 schools selected from the Education Bureau's official list. In the second stage, parents were recruited within these schools based on consent and eligibility, constituting a convenience sample. Parents completed standardized questionnaires assessing sociodemographic characteristics, perceived effectiveness of transition practices, school readiness, and child mental health. Regression analyses examined associations among these variables.

**Results:**

Mothers reported higher school readiness scores than fathers. Parental perceptions of curriculum- and administration-focused transition practices were positively associated with multiple school readiness subscales, whereas home–school collaboration practices were linked only to self-management. Emotional and behavioral development emerged as the strongest predictor of reduced depression and anxiety symptoms. Moderation analyses indicated stronger associations between curriculum- and administration-focused practices and general preparedness among low-income families and parents with secondary education or below. Sensitivity analyses using bootstrapping confirmed the robustness of these findings. After Bonferroni corrections, only the interaction between general preparedness and parent gender in predicting mental health outcomes remained significant.

**Conclusions:**

Findings underscore the importance of tailoring transition practices to the needs of socioeconomically disadvantaged families to promote equitable school readiness and mental health outcomes.

## Highlights

- Empirical evidence on school readiness and mental health outcomes in Hong Kong, adding insights from an underrepresented Chinese context
- Emotional and behavioral development identified as the strongest predictor of reduced depression and anxiety symptoms in early childhood
- Interaction effects involving parents with diploma-level education and school readiness or mental health outcomes remained robust across sensitivity analyses
- Statistically significant interaction between general preparedness and parent gender in predicting child mental health outcomes

## 1 Introduction

The transition from kindergarten to primary school (K-P) represents a critical developmental milestone for children (1, 2). This transition requires adaptation to new learning environments, curricula, school routines, pedagogical approaches, and interpersonal relationships with teachers and peers (3–5). While many children manage this transition successfully, research indicates that up to 48% of children encounter adjustment difficulties (6), and about 9.2% of Australian parents report that their children resist attending school (7). Such challenges can precipitate stress and psychological distress, manifesting as anxiety, sleep disturbances, and emotional dysregulation (8–10). Importantly, these difficulties are not transient; they carry lifelong consequences for academic achievement and mental health that extend into adolescence and adulthood (11). Thus, the K-P transition must be recognized not merely as an entry point into formal education, but as a pivotal developmental juncture with profound academic, psychological and societal implications, underscoring the urgent need for coordinated support from educators, families, and communities (12–15).

Within Chinese society, such as Hong Kong, where academic emphasis and competition are deeply embedded and closely tied to familial aspirations and cultural expectations (16, 17), the K-P transition can be especially challenging and warrants further scholarly investigation. Morawska et al. highlighted “a gap in our understanding of parents’ views, experiences, and preferences about their child’s transition to school, and the parent voice is largely missing from existing research” [ (18), p. 2]. Likewise, a systematic review by Eckert et al. identified only seven studies on transition programs, most of which focused on teachers’ perceptions of transition-related processes and practices (19). Fane et al. similarly noted that most instruments assessing children's health and wellbeing during school transition were completed by teachers, educators, or health practitioners rather than parents (20). Collectively, these findings underscore the limited attention given to parental perspectives in the literature.

Against this background, this study aims to address these gaps by (i) examining the relationships between Chinese parents’ perceptions of transition practices and children's school readiness, (ii) investigating the association between school readiness and child mental health outcomes, and (iii) exploring the moderating role of sociodemographic factors in these relationships.

This investigation is warranted given the limited attention to parental perspectives and the heightened challenges of the K–P transition in Chinese society, where academic demands are heavily stressed. By examining parental perceptions of transition practices and school readiness in relation to child mental health, and by considering sociodemographic moderators, this study contributes new evidence from non-Western context that extends the existing literature. Importantly, school readiness intersects with issues of educational equity, as children from socioeconomically disadvantaged families often encounter additional barriers, and their mental health and wellbeing have been shown to be particularly at risk (21). These findings can inform educators and practitioners of the need to tailor interventions to diverse family contexts and to ensure equitable support for children's adjustment. Overall, the study advances understanding of Chinese parents' perspectives on school transition practices, informs culturally responsive approaches, and contributes to addressing issues of equity in child development.

### 1.1 Child mental health

Child **mental health** is the primary outcome of this study, recognized as a core dimension of healthy development with enduring consequences across the life course. For instance, in a cohort of more than 34,000 children, Thomson et al. found that poor socio-emotional functioning at school entry was associated with more than double the odds of subsequent mental health problems, including depression, anxiety, and conduct disorder by age 14 (22).

Evidence consistently shows that early developmental competencies, particularly socio-emotional skills, self-regulation skills, language abilities, and approaches to learning, are strongly linked to later resilience, reduced behavioral difficulties, and lower risks of psychopathology (23–25). These competencies also underpin school readiness, directly linking children's preparedness for formal schooling to their mental health and long-term well-being.

Taken together, these findings underscore child mental health as a crucial developmental outcome that influences trajectories across childhood and adolescence. Building on this evidence, the present study investigates how parental perceptions of transition practices influence children's school readiness, which ultimately impacts their mental health.

### 1.2 School readiness

**School readiness** is a multifaceted construct comprising three interrelated yet conceptually distinct aspects: ready children, ready schools, and ready family and communities (26). This study focuses on child school readiness, defined as the child's ability to function successfully in the school context, reflecting developmental competencies across multiple domains necessary for adjustment to formal schooling (27). The U.S. Department of Education identifies five domains central to school readiness: language and cognitive development, approaches to learning, general knowledge, physical well-being and motor development, and socio-emotional development. In the Chinese context, Lau et al. developed the Chinese Readiness for School Scale (CRSS), which operationalizes readiness across six domains: general preparedness, general knowledge and cognitive development, social development, behavioral and emotional development, approaches to learning, and physical development (28), aligned with international frameworks (29).

Among these domains, socio-emotional competence is particularly influential, fostering perseverance, supporting positive peer relationships, and reducing risks of academic failure and psychopathology (23, 24). Longitudinal evidence further shows that socio-emotional skills acquired in kindergarten predict well-being years later (15).

Together, these domains underscore school readiness as a set of developmental competencies that not only enable optimal school performance but also form the foundation for successful adjustment and mental health outcomes. This linkage sets the stage for examining how parental perceptions of transition practices influence children's school readiness and, in turn, their mental health.

### 1.3 Transition practices

**Transition practices** encompass a range of organized activities designed to connect kindergartens and primary schools, fostering collaboration among schools and families to support the K–P transition (30, 31). These practices aim to strengthen children's preparedness for school, which is a strong predictor of later academic attainment, health outcomes, and personal success into adulthood (32). Globally, diverse transition practices have been implemented to facilitate young children's move from early childhood education (ECE) to primary school, though their design and emphasis vary considerably across cultural, educational, and policy contexts.

In Western settings, common practices include pre-term school visits and orientations to familiarize children with new environments, routines, and staff (4, 33); information-sharing with parents through visits or brochures (34, 35); shortened school days at the beginning of the academic year (36); and peer mentoring programs in which older students support younger peers. These practices have been associated with improved adjustment during the first year of school, reduced behavioral problems, and enhanced social and academic performance (37, 38).

Conversely, transition practices in Hong Kong and mainland China tend to prioritize academic preparedness, emphasizing pre-reading skills (e.g., letter recognition), early numeracy (e.g., counting and number recognition), curriculum continuity between ECE and primary settings, and parental briefings to align expectations (39, 40). These emphases reflect a broader societal orientation toward academic excellence and competitiveness (41, 42).

Despite these differences, both Western and Chinese contexts underscore the important role of collaboration among educators, families, and communities in supporting successful transitions. Building on these diverse practices, parental perceptions become critical in determining how effectively transition practices support children's school readiness.

### 1.4 Influence of parental perceptions of transition practices on school readiness and mental health

**Parental perceptions of transition practices** play a pivotal role in shaping children's school readiness (43). When parents perceive these practices as effective, they are more likely to provide sustained support that enhances children's academic and social development, fostering confidence and competence across readiness domains. In this way, parental perceptions function not merely as attitudes but as active mechanisms influencing preparedness for school. Hence, we hypothesize that higher parental perceptions of the effectiveness of transition practices will be positively associated with stronger school readiness.

Parental perceptions of school readiness likewise shape developmental trajectories. When parents consider their child well prepared, they tend to experience reduced anxiety, provide consistent support, and foster nurturing relationships that can protect children's mental health. Conversely, low parental perceptions may heighten stress, undermine engagement, and exacerbate vulnerabilities. Accordingly, we hypothesize that higher parental perceptions of school readiness will be negatively associated with poor child mental health outcomes.

### 1.5 Moderating role of sociodemographic factors

Grounded in Bronfenbrenner's Ecological Systems Theory (44), which has been increasingly applied in school transition research (45), this study conceptualizes **sociodemographic factors** as proximal contextual moderators that shape the dynamic interplay between parental perceptions of transition practices, children's school readiness, and mental health outcomes. Within the microsystem, parental roles and caregiving patterns exert a direct influence on children's developmental experiences. In two-parent households, mothers often continue to serve as primary caregivers (46), particularly in time-intensive domains such as school-related activities (47). These gender-specific caregiving patterns may result in differing emphases on academic vs. behavioral preparedness, thereby shaping parental perceptions and the type of support provided during transitional periods.

Socioeconomic factors such as parental education and family income further determine the resources, expectations, and opportunities available to families. Parental education influences parenting practices and the quality of the home learning environment (48, 49), while family income affects access to educational materials and structured support services (50). Children from socioeconomically disadvantaged backgrounds may be more vulnerable to transition-related stress (51), yet also more responsive to targeted interventions. Evidence indicates that maternal education is more strongly associated with academic outcomes (52), whereas family income predicts behavioral development (53).

Importantly, sociodemographic factors not only predict developmental outcomes but also moderate the extent to which parents can translate beliefs into meaningful support (54). Cook et al. noted that “while children in the same schools may be given similar opportunities for transition supports, it does not mean that all families are able to access them in the same way” [(55), p. 743]. Structural constraints, such as inflexible work schedules, financial limitations, and lower educational attainment, can restrict parental engagement.

Despite extensive global research on school transitions, few studies have examined how sociodemographic factors moderate the relationship between parental perceptions of transition practices and child mental health outcomes within Chinese cultural contexts. This gap is particularly salient in Hong Kong, where competitive academic environments, the high-stakes nature of early assessments, and normative expectations around parental involvement place heightened demands on families (14). A deeper understanding of these moderating effects is essential for designing equitable, culturally responsive transition practices that support children across diverse family backgrounds.

### 1.6 Study hypotheses

This study examined the relationships between parental perceptions of transition practices, school readiness, and children's mental health within the Hong Kong context. It also explored the moderating role of sociodemographic factors, focusing on how parental characteristics such as education and income influence the strength and direction of these associations. The following hypotheses were tested:

**H1:** Higher parental perceptions of the effectiveness of transition practice will be positively associated with stronger school readiness outcomes.
**H2:** Higher parental perceptions of school readiness will be negatively associated with poor child mental health outcomes.
**H3:** Sociodemographic factors will moderate the relationship between the perceived effectiveness of transition practices and child mental health outcomes.

Drawing on Bronfenbrenner's Ecological Systems Theory (44), we posit that sociodemographic factors such as family income and parental education moderate the relationship between parental perceptions of the effectiveness of transition practice and child mental health outcomes. These factors influence children's access to informal support systems and coping resources. Specifically, for children from lower-income households or with less-educated parents, effective transition practices may serve a compensatory function by offering structure and emotional support that might otherwise be limited, thereby exerting a stronger positive influence on mental health. In contrast, children from more advantaged backgrounds may already benefit from many protective resources that buffer transition-related stress regardless of perceived transition support, resulting in a weaker association between transition practices and mental health outcomes.

**H4:** Sociodemographic factors will moderate the relationship between perceived school readiness and child mental health outcomes.

We anticipate that high perceived school readiness will be more strongly associated with positive mental health outcomes among children from socioeconomically disadvantaged backgrounds, where readiness may reflect resilience and preparedness in the face of structural barriers. In contrast, for children from more advantaged families, school readiness may be normative and therefore, less predictive of mental health outcomes, given their broader access to developmental supports and protective resources.

**Figure 1:**
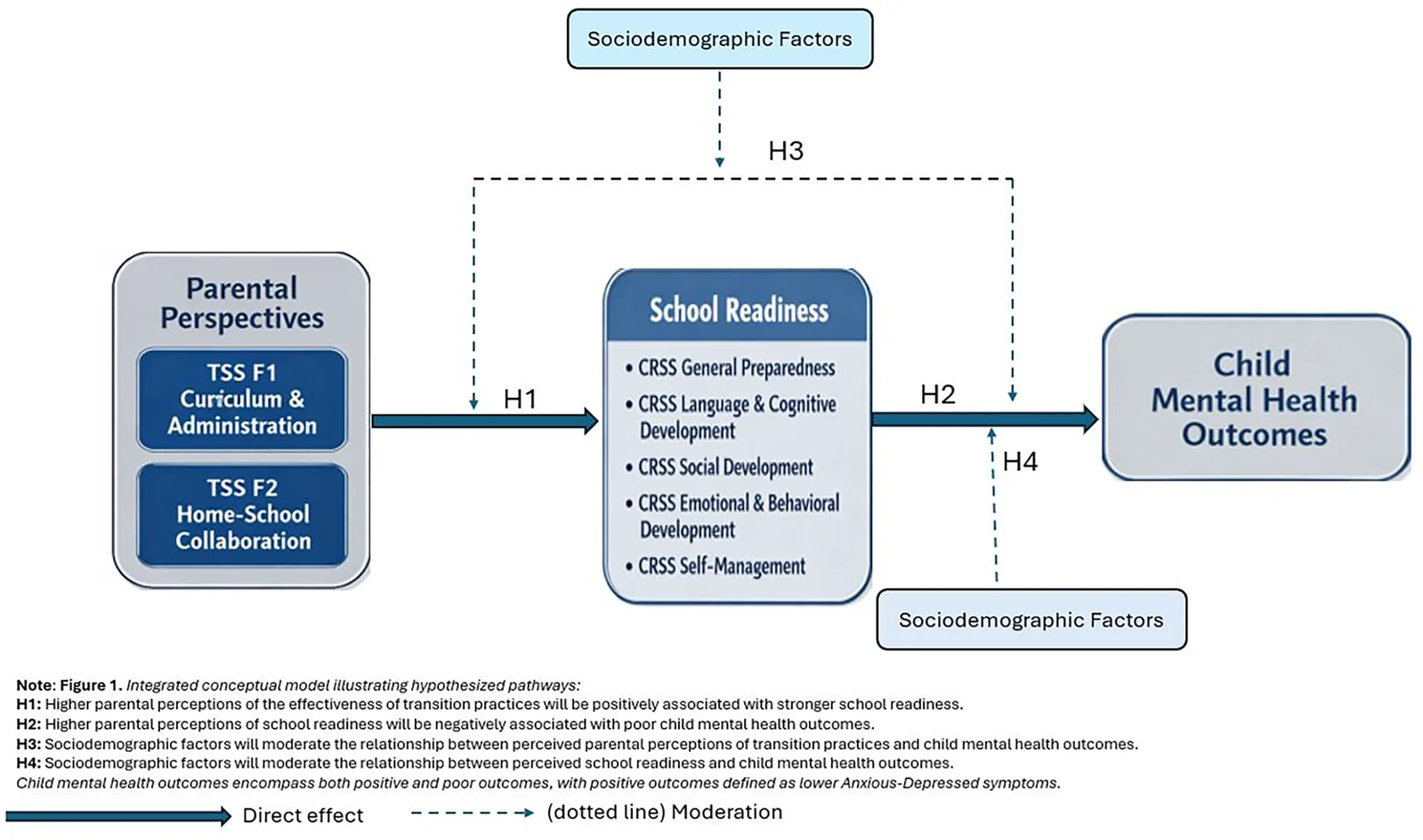
Proposed integrated conceptual model.

Figure 1 presents the conceptual model illustrating these four hypothesized relationships.

## 2 Method

### 2.1 Study design

This study forms part of a larger longitudinal, mixed methods project, employing a research design whose longitudinal component is consistent with approaches commonly found in the existing literature [e.g., (56)]. Conducted between 2017 and 2018, the study examined the impact of parental perceptions of transition practices on children's school readiness and mental health. We report findings from the cross-sectional dataset collected during the first wave of the longitudinal study, in which data were gathered at two time points separated by three months.

### 2.2 Recruitment of schools and participants

Cross-sectional data were collected through a two-stage sampling process. In the first stage, primary schools were stratified by region (Hong Kong Island, Kowloon, and the New Territories), and a random sample of 21 schools was selected from the Education Bureau's official list, with international schools excluded. Invitation emails outlining the study objectives and procedures were sent to principals, followed by reminder phone calls if no response was received within one week. In the second stage, schools that agreed to participate distributed consent forms to parents of Primary One students. Within these schools, parent participation was voluntary and based on consent and eligibility, constituting a convenience sample.

Eligibility criteria required parents to be of Chinese ethnicity, aged 18 years or above, and able to read Chinese. Those who provided written consent completed an anonymous, self-administered questionnaire at baseline (T1, towards the end of Primary one), and again at follow-up (T2, at the beginning of Primary Two), with a three-month interval between data collections.

To ensure confidentiality, questionnaires were distributed and returned in sealed envelopes via the students at the participating schools, and collected by the research assistant on designated dates. Identical procedures were followed at both T1 and T2. All research procedures were reviewed and approved by the Human Research Ethics Committee (HREC) of the corresponding author's university.

### 2.3 Participant information

A total of 278 parents were recruited for the study. After excluding cases with missing gender data, the final sample comprised 259 parents from 21 primary schools located across Hong Kong Island, Kowloon, and the New Territories.

The parent participants were predominantly female (82.6%), with a mean age of 38.99 years (SD = 5.71). In terms of educational attainment, 55.6% had completed secondary school or below, while 23.6% held a bachelor's degree or higher. The majority were married (93.8%), and over 60% resided in households with four or more members. Regarding household composition, 24.4% reported employing a domestic helper. More than half had two children, while 32.2% indicated that the Primary One child was an only child. Employment status varied: 36.0% of participants worked full-time, and 42.6% identified as housewives or househusbands. Reported monthly household income showed that 36.6% of families earned less than HK$19,999. Table 1 presents the descriptive statistics for the sociodemographic information of the parent participants.

**Table 1:** Sociodemographic characteristics and mean scores of all study variables by parent gender (*n* = 259).

Variables	Male	Female	Total	*t*-test *p*-value	Cohen’s *d*
Age, mean (SD)	43.23 (7.96)	38.04 (4.59)	38.99 (5.71)		
Gender, *n* (%)					
Male			45 (17.4%)		
Female			214 (82.6%)		
Education level, *n* (%)					
Secondary school or below	21 (46.7%)	123 (57.5%)	144 (55.6%)		
Diploma or Associate degree	8 (17.8%)	46 (21.5%)	54 (20.8%)		
Bachelor or above	16 (35.6%)	45 (21.0%)	61 (23.6%)		
Marital status, *n* (%)					
Married	44 (97.8%)	197 (92.9%)	241 (93.8%)		
Cohabiting	0 (0.0%)	2 (0.9%)	2 (0.8%)		
Separated	0 (0.0%)	1 (0.5%)	1 (0.4%)		
Divorced	1 (2.2%)	8 (3.8%)	9 (3.5%)		
Widowed	0 (0.0%)	2 (0.9%)	2 (0.8%)		
Others	0 (0.0%)	2 (0.9%)	2 (0.8%)		
Domestic helper at home, *n* (%)					
Yes	14 (31.1%)	49 (23.0%)	63 (24.4%)		
No	31 (68.9%)	164 (77.0%)	195 (75.6%)		
Number of household family members (excluding domestic helpers), *n* (%)					
1	0 (0.0%)	3 (1.4%)	3 (1.2%)		
2	3 (6.7%)	18 (8.5%)	21 (8.2%)		
3	9 (20.0%)	57 (26.9%)	66 (25.7%)		
4	20 (44.4%)	97 (45.8%)	117 (45.5%)		
5 or above	13 (28.9%)	37 (17.5%)	50 (19.5%)		
Total number of children, *n* (%)					
1	15 (33.3%)	70 (32.7%)	85 (32.8%)		
2	23 (51.1%)	122 (57.0%)	145 (56.0%)		
3	5 (11.1%)	20 (9.3%)	25 (9.7%)		
4 or above	2 (4.4%)	2 (0.9%)	4 (1.5%)		
Birth order of the child enrolled in Primary One, *n* (%)					
Only child	14 (31.1%)	69 (32.4%)	83 (32.2%)		
Oldest child	19 (42.2%)	70 (32.9%)	89 (34.5%)		
Middle child	2 (4.4%)	10 (4.7%)	12 (4.7%)		
Youngest child	10 (22.2%)	64 (30.0%)	74 (28.7%)		
Employment status, *n* (%)					
Full time (≥ 40 h/week)	35 (77.8%)	58 (27.2%)	93 (36.0%)		
Part time (≤ 40 h/ week)	3 (6.7%)	43 (20.2%)	46 (17.8%)		
Unemployed	4 (8.9%)	1 (0.5%)	5 (1.9%)		
Retired	0 (0.0%)	4 (1.9%)	4 (1.6%)		
Househusband/Housewife	3 (6.7%)	107 (50.2%)	110 (42.6%)		
Monthly household income, *n* (%)					
$19,999 or below	22 (48.9%)	71 (34.0%)	93 (36.6%)		
$20,000-$39,999	12 (26.7%)	76 (36.4%)	88 (34.6%)		
$40,000 or above	11 (24.4%)	62 (29.7%)	73 (28.7%)		
TSS F1, mean (SD)	4.18 (0.49)	4.31 (0.53)	4.29 (0.52)	0.116	0.259
TSS F2, mean (SD)	4.12 (0.53)	4.29 (0.55)	4.26 (0.55)	0.071	0.298
CRSS	3.72 (0.53)	4.02 (0.51)	3.96 (0.54)	<0.001***	0.593
CRSS General Preparedness, mean (SD)	3.79 (0.61)	4.10 (0.56)	4.05 (0.58)	0.001**	0.541
CRSS Language and Cognitive Development, mean (SD)	3.72 (0.68)	4.05 (0.56)	3.99 (0.60)	<0.001***	0.565
CRSS Social Development, mean (SD)	3.76 (0.55)	4.03 (0.54)	3.98 (0.55)	0.003**	0.492
CRSS Emotional and Behavioral Development, mean (SD)	3.81 (0.66)	4.04 (0.61)	4.00 (0.63)	0.022*	0.378
CRSS Self-management, mean (SD)	3.26 (0.86)	3.71 (0.85)	3.63 (0.86)	0.001**	0.528
A/D-CBCL, mean (SD)	5.98 (3.89)	5.56 (4.30)	5.63 (4.23)	0.544	0.100

Mothers reported significantly higher school readiness scores than fathers across all domains, with effect sizes ranging from *d* = 0.378 to 0.593, indicating small to medium effects. No significant gender differences were observed in reported transition practices or in children’s mental health symptoms.

Figures were computed after excluding cases with missing data.

TSS F1, Transition Strategies Scale Factor 1 (Curriculum and Administration); TSS F2, Transition Strategies Scale Factor 2 (Home-School Collaboration); CRSS, Chinese Readiness for School Scale; A/D-CBCL, Anxious/Depressed subscale of Child Behavior Checklist.

*p < .05, **p < .01, ***p < .001.

### 2.3 Measures

#### 2.3.1 Socio-demographics

The questionnaire included a section on participants' demographic information, covering variables such as age, gender, marital status, educational attainment, number of children, birth order of the child currently enrolled in Primary One, monthly household income, and the presence of foreign domestic helpers, a common arrangement among middle- and higher-income families in Hong Kong.

For the purpose of data analysis, parental education was categorized into three groups: (i) completion of secondary school or below, (ii) diploma or associate degree, and (iii) bachelor’s degree or above. Monthly household income was classified into three tiers based on official statistics from the Hong Kong Census and Statistics Department. The median monthly household income in 2017 was HK$26,500, which informed the categorization (57). Households earning HK$19,999 or below were classified as low income, those earning HK$20,000–39,999 as middle income, and those earning HK$40,000 or above as high income. This three-tier classification reflects the local income distribution in Hong Kong at the time of data collection.

#### 2.3.2 Parents' perceptions of transition practices

Parents' perceptions of school transition practices were assessed using the Transition Strategies Scale (TSS), which evaluates the perceived effectiveness of various transition practices implemented by primary schools to support children's adjustment from kindergarten to Primary One.

The TSS was developed through a systematic four-phase process: First, items were generated based on a comprehensive literature review of school transition practices. Second, focus group interviews were conducted with parents to gather qualitative insights. Third, an expert panel reviewed the items for content validity. Finally, statistical analyses were performed to assess reliability and validity.

Parent participants rated each item on a five-point Likert scale ranging from 1 (“strongly disagree”) to 5 (“strongly agree”), with higher scores indicating greater perceived effectiveness of the strategies.

Exploratory factor analysis (EFA) and confirmatory factor analysis (CFA) supported a two-factor structure, based on parent data collected at two time points. Factor 1 (F1): Curriculum and Administration encompass school-based practices designed to ease children's adaptation to the primary school environment. Example items include “Arranging senior students to help Primary One (P1) students”, “Visiting P1 students at recess or lunch, accompanying them while eating or telling stories to facilitate campus life adaptation”, “Reducing students’ learning pressure by setting reasonable learning expectations”.

Factor 2 (F2): Home-School Collaboration involves parental engagement and communication strategies to support children during the transition. Example items include “Providing individual talks with parents to enhance understanding of the school’s expectations”, “Creating opportunities for home-school collaboration (e.g., ‘lunch aunts,’ ’story parents’) to improve parental awareness of their children’s school experience”

**Figure 2:**
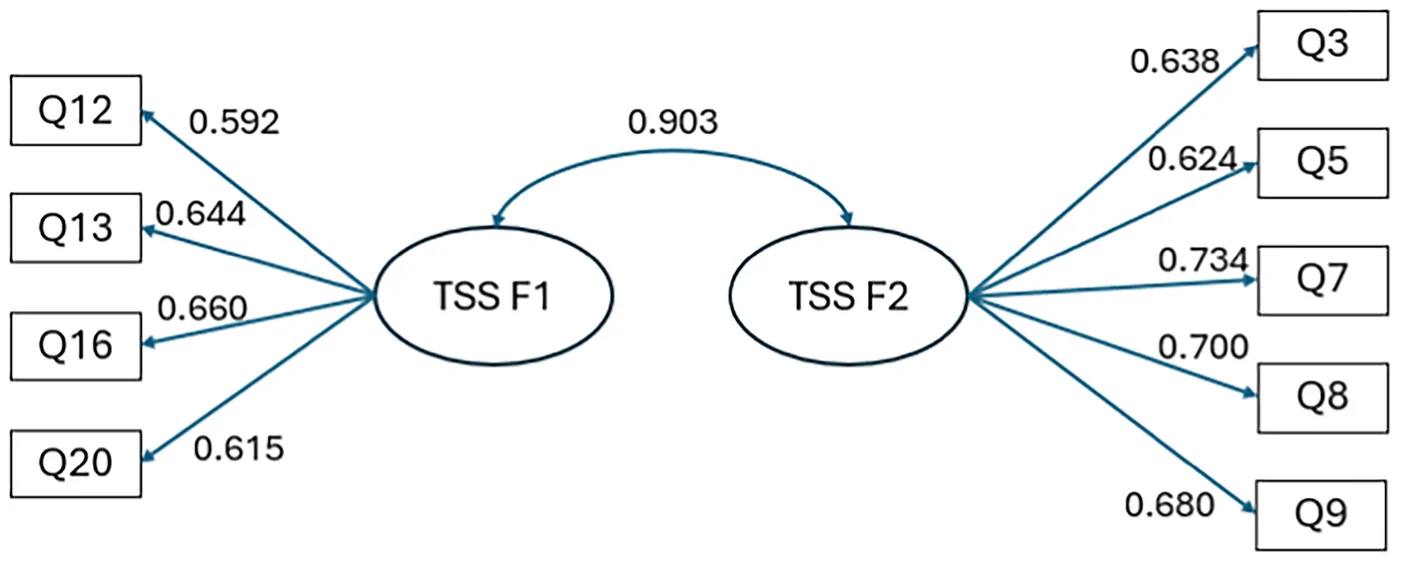
Confirmatory factor analysis supporting a two-factor structure.

The scale demonstrated strong psychometric properties. Internal consistency reliability was assessed using Cronbach’s alpha, which confirmed satisfactory reliability for both factors (F1: Cronbach's *α* = 0.714; F2: Cronbach's *α* = 0.759). F1 consists of four items, while F2 comprises five items. The specific items associated with each factor are illustrated in Figure 2.

Test-retest reliability over a three-month interval indicated stable responses. CFA was conducted using the maximum likelihood estimator on the hypothesized two-factor model. Construct validity was further supported by model fit indices from CFA, including CFI, TLI, RMSEA, which confirmed the adequacy of the factor structure. The model fit was excellent: *χ*²(26) = 49.32, *p* = .004; CFI = .98; TLI = .98; RMSEA = .04 [90% CI (.02,.06)]; SRMR = .03. All items loaded significantly onto their respective factors, with standardized loadings ranging from 0.59 to 0.73. The two factors were strongly correlated (*r* = .90). These results support the construct validity and the adequacy of the two-factor structure for the measurement (see Figure 2).

#### 2.3.3 School readiness

Children's readiness for school was assessed using the Chinese Readiness for School Scale (CRSS) (28). The scale consists of 35 items grouped into six subscales: (1) General Preparedness for School (e.g., “able to follow the instructions”, “willing to learn and take initiative”), (2) Language and Cognitive Development (e.g., “can make simple Chinese sentences”, “able to retell stories”), (3) Social Development (e.g., “able to share toys with other children”, “like making friends”), (4) Emotional and Behavioral Development (e.g., “does not push other children”, “does not say impolite words”), (5) Self-management (e.g., “able to tidy up toys by him/herself”, “can bring the required supplies”), and (6) Motor Development (e.g., “able to use a spoon and chopsticks”, “able to use scissors to cut paper”). In the current study, motor development was excluded to maintain construct specificity, as the focus was on domains most salient to parental perceptions, specifically academic and behavioral preparedness.

The CRSS has been established as a valid and culturally reliable measure in Hong Kong educational contexts (28). In the present study, the Cronbach's alpha of CRSS and its subscales were: CRSS (Cronbach's *α* = 0.948), CRSS General Preparedness (Cronbach's *α* = 0.866), Language and Cognitive Development (Cronbach's *α* = 0.844), Social Development (Cronbach's *α* = 0.836), Emotional and Behavioral Development (Cronbach's *α* = 0.808), and Self-Management (Cronbach's *α* = 0.840).

#### 2.3.4 Mental health

Children's mental health was assessed using the Anxious/Depressed (A/D) subscale of the Child Behavior Checklist [CBCL (58)], which captures parents' perceptions of their child's anxiety and depressive symptoms. Each item on the A/D subscale is rated on a three-point Likert scale: 0 (“Not True”), 1 (“Somewhat or Sometimes True”), or 2 (“Very True or Often True”). The Chinese version of the A/D-CBCL has demonstrated strong internal consistency and criterion validity in previous studies [e.g., (59)]. In the current study, the subscale showed good internal reliability, with Cronbach's *α* = 0.811.

### 2.4 Statistical analysis

All statistical analyses were conducted using IBM SPSS Statistics (Version 27.0). Statistical significance was set at *p* < 0.05. Reliability tests were performed to assess the internal consistency of the scales, with results reported using Cronbach's alpha. Descriptive statistics were presented for parents' demographic characteristics, and means and standard deviations were calculated for all study variables, disaggregated by parent gender.

Pearson correlation coefficients were computed to examine bivariate associations among variables. All continuous variables were mean-centered prior to regression analyses to reduce multicollinearity in interaction terms.

Missing data were addressed using complete-case analysis, with cases containing missing values on key study variables excluded from the relevant analyses. The normality of all continuous study variables was assessed using Kolmogorov–Smirnov tests, supplemented by visual inspection of histograms and normal Q-Q plots.

To test H1, multiple linear regression analyses were conducted to examine whether the two factors of TSS predicted the five domains of CRSS.

To test H2, multiple linear regression was used to examine whether CRSS subscales predicted children's mental health outcomes, as measured by the A/D-CBCL. In this model, A/D-CBCL was entered as the dependent variable, with CRSS subscales included as independent variables.

For H3 and H4, moderation analyses were performed using hierarchical multiple regression. Each model tested whether sociodemographic factors moderated the effect of TSS on CRSS or the effect of CRSS on A/D-CBCL. Interaction terms between the independent predictors and sociodemographic factors were computed and analyzed to assess moderation effects.

## 3 Results

### 3.1 Descriptive statistics and correlations

Table 1 presents the descriptive statistics of sociodemographic characteristics and the mean scores for various scales by parent gender. Significant gender differences were observed across the CRSS and all five of its subscales. Compared to fathers, mothers scored higher on CRSS (M = 4.02, SD = 0.51 vs. M = 3.72, SD = 0.53), CRSS General Preparedness (M = 4.10, SD = 0.56 vs. M = 3.79, SD = 0.61), Language and Cognitive Development (M = 4.05, SD = 0.56 vs. M = 3.72, SD = 0.68), Social Development (M = 4.03, SD = 0.54 vs. M = 3.76, SD = 0.55), Emotional and Behavioral Development (M = 4.04, SD = 0.61 vs. M = 3.81, SD = 0.66), and Self-Management (M = 3.71, SD = 0.85 vs. M = 3.26, SD = 0.86). There were no significant differences between genders in TSS F1, F2 and A/D-CBCL.

Formal tests indicated statistically significant departures from normality for several observed variables. However, given the sample size of 259 and the established robustness of Pearson correlation and linear regression to moderate non-normality, the planned parametric analyses were retained. The assumptions of linear regression were evaluated at the model level through inspection of residual distributions and diagnostic statistics. In addition, sensitivity analyses using bootstrapping were conducted to assess the robustness of the findings under reduced reliance on normality assumptions.

As shown in Table 2, both F1 and F2 of the TSS exhibited small to moderate positive correlations with all CRSS subscales (*r* = 0.213–0.329, all *p* < .001), indicating that higher perceived effectiveness of transition practices was associated with greater school readiness. In contrast, CRSS subscales were negatively correlated with A/D-CBCL scores (*r* = –0.183 to −0.380, *p* < .001), suggesting that higher levels of school readiness were associated with fewer internalizing symptoms in children.

**Table 2:** Pearson’s correlation among TSS, CRSS subscales, and A/D-CBCL.

	Variables	2	3	4	5	6	7	8
1	TSS F1	.684^a^	.327^a^	.306^a^	.329^a^	.287^a^	.213^a^	−.090
2	TSS F2		.298^a^	.289^a^	.268^a^	.253^a^	.256^a^	−.078
3	CRSS General Preparedness			.833^a^	.718^a^	.614^a^	.713^a^	−.255^a^
4	CRSS Language and Cognitive Development				.638^a^	.596^a^	.655^a^	−.190^a^
5	CRSS Social Development					.733^a^	.565^a^	−.329^a^
6	CRSS Emotional and Behavioral Development						.552^a^	−.380^a^
7	CRSS Self-management							−.183^a^
8	A/D-CBCL							

Strong correlations were observed between TSS F1 and TSS F2 (*r* = .684), as well as between CRSS General Preparedness and CRSS Emotional and Behavioral Development (*r* = .614 −.833). Mental health symptoms were most strongly associated with CRSS Emotional and Behavioral Development (*r* = −.380).

TSS F1, Transition Strategies Scale Factor 1 (Curriculum and Administration); TSS F2, Transition Strategies Scale Factor 2 (Home-School Collaboration); CRSS, Chinese Readiness for School Scale; A/D-CBCL, Anxious/Depressed subscale of Child Behavior Checklist.

a Correlation is significant at the 0.01 level (2-tailed).

### 3.2 Main effects analyses on school readiness

TSS F1 significantly predicted four out of the five CRSS subscales, except Self-Management. Specifically, F1 was significantly associated with General Preparedness (*B* = .263, *p* = .004), Language and Cognitive Development (*B* = .238, *p* = .012), Social Development (*B* = .293, *p* = .001), and Emotional and Behavioral Development (*B* = .261, *p* = .009). However, F1 was not significantly related to Self-management (*B* = .118, *p* = .392). In contrast, F2 emerged as a significant predictor exclusively for Self-management (*B* = .332, *p* = .012). Together, these findings partially suggest that while parental perceptions of school transition practices represented by F1 are positively associated with four subscales of CRSS, F2 is only associated with Self-Management, indicating varied patterns of influence across developmental domains (Table 3).

**Table 3:** Regression analysis of TSS predicting children’s readiness for school scale (CRSS).

Predictors	*B*	*SE B*	*β*	*T*	*P*	Partial *η*^2^
Predicting CRSS General Preparedness						
Constant	0.005	0.034	—	0.156	0.876	0.000
TSS F1	0.263	0.090	0.233	2.911	0.004**	0.031
TSS F2	0.148	0.086	0.137	1.714	0.088	0.011
Predicting CRSS Language and Cognitive Development						
Constant	0.005	0.036	—	0.144	0.886	0.000
TSS F1	0.238	0.094	0.203	2.516	0.012*	0.023
TSS F2	0.168	0.090	0.150	1.859	0.064	0.013
Predicting CRSS Social Development						
Constant	0.005	0.032	—	0.158	0.874	0.000
TSS F1	0.293	0.086	0.275	3.428	0.001**	0.043
TSS F2	0.079	0.082	0.078	0.971	0.333	0.004
Predicting CRSS Emotional and Behavioral Development						
Constant	0.005	0.037	—	0.135	0.893	0.000
TSS F1	0.261	0.099	0.214	2.630	0.009**	0.026
TSS F2	0.123	0.095	0.106	1.301	0.194	0.006
Predicting CRSS Self-management						
Constant	0.005	0.052	—	0.094	0.925	0.000
TSS F1	0.118	0.138	0.070	0.857	0.392	0.003
TSS F2	0.332	0.131	0.207	2.524	0.012*	0.024

A regression analysis examining transition practices (TSS) as predictors of the Children’s Readiness for School Scale (CRSS) revealed that TSS F1 (curriculum- and administration-focused) predicted four readiness domains (*β* = .203−.275), while TSS F2 (home-school collaboration-focused) uniquely predicted self-management (*β* = .207). All effects reflected small effect sizes.

TSS F1, Transition Strategies Scale Factor 1 (Curriculum and Administration); TSS F2, Transition Strategies Scale Factor 2 (Home-School Collaboration); CRSS, Chinese Readiness for School Scale.

*p < .05, **p < .01.

### 3.3 Main effects analyses on mental health

In the full regression model including all CRSS subscales, only Emotional and Behavioral Development significantly predicted child mental health outcomes (*B* = –2.268, *p* < 0.001). This indicated that higher levels of emotional and behavioral readiness were associated with fewer depressive symptoms and lower anxiety. The relationship remained consistent across nested models, with Emotional and Behavioral Development maintaining a robust predictive role in all reduced models (*B* = –2.595, *p* < 0.001). These findings provide strong support for the critical role of social-emotional readiness in promoting children's mental health (Table 4).

**Table 4:** Regression analysis of children’s readiness for school scale (CRSS) predicting A/D-child behavior checklist (CBCL).

Predictors	*B*	*SE B*	*β*	*T*	*P*	Partial *η*^2^
Full model						
Constant	0.015	0.245	—	0.060	0.952	0.000
CRSS General Preparedness	−1.064	0.889	−0.143	−1.197	0.232	0.006
CRSS Language and Cognitive Development	1.126	0.750	0.156	1.501	0.135	0.009
CRSS Social Development	−0.932	0.750	−0.119	−1.243	0.215	0.006
CRSS Emotional and Behavioral Development	−2.268	0.592	−0.332	−3.829	<0.001***	0.054
CRSS Self-management	0.313	0.413	0.063	0.757	0.450	0.002
Reduced model						
Constant	0.017	0.246		0.069	0.945	0.000
CRSS Emotional and Behavioral Development	−2.595	0.391	−0.380	−6.645	<0.001***	0.144

A regression analysis examining the predictive value of the Children’s Readiness for School Scale (CRSS) on the Anxiety/Depression subscale of the Child Behavior Checklist (CBCL) revealed that only the Emotional and Behavioral Development domain significantly predicted fewer anxiety/depression symptoms (*β* = −.380, *p* < .001), indicating a large effect size. Other readiness domains were not significantly associated with anxiety/depression outcomes.

CRSS, Chinese Readiness for School Scale; A/D-CBCL, Anxious/Depressed subscale of Child Behavior Checklist.

***p < .001.

### 3.4 Moderation analyses by parent age

Parent age did not significantly moderate the relationship between TSS and CRSS, as all interaction terms between age and TSS were non-significant. However, a significant interaction was found between CRSS Social Development and parent age (*B* = .152, *p* = .043). The pattern indicated that higher levels of CRSS Social Development were strongly protective against depressive and anxiety symptoms among younger parents, less protective among older parents, and, for much older parents, unexpectedly associated with more internalizing symptoms. This suggests that the protective influence of social development on children's mental health diminishes with parental age and may even reverse in later parenthood.

### 3.5 Moderation analyses by parent gender

Similarly, parent gender did not significantly moderate the relationship between TSS and CRSS, with all interaction terms between gender and TSS remaining non-significant. However, a significant interaction emerged between CRSS General Preparedness and parent gender in relation to children's mental health outcomes (*B* = 2.718, *p* = .016). Specifically, higher levels of General Preparedness were associated with lower A/D-CBCL scores in mothers' responses, suggesting fewer internalizing symptoms. In contrast, this association was weaker among fathers, indicating that General Preparedness was less strongly linked to reductions in itigating children's mental health symptoms. All other interaction terms between CRSS subscales and gender were non-significant.

### 3.6 Moderation analyses by household income

Household income significantly moderated the effect of TSS F1 on CRSS General Preparedness, with a stronger positive association observed among low-income families (*B* = .335, *p* = .046). This indicates that in households with lower income, higher TSS F1 scores are more strongly linked to greater General Preparedness. No other significant interaction effects were found for household income, suggesting that TSS did not differentially affect other CRSS subscales across income levels. Furthermore, household income did not moderate the relationship between parental perceptions of school readiness and children's mental health outcomes, indicating consistent effects of school readiness on mental health across income groups.

### 3.7 Moderation analyses by parental education

Regarding parental education, several significant interaction effects between TSS factors and education level were observed. Specifically, the association between TSS F1 and CRSS General Preparedness was significantly stronger among parents with secondary education or below (*B* = .308, *p* = .043) compared to those with higher education attainment. A similar interaction effect was observed for Emotional and Behavioral Development, where the effect of TSS F1 was more pronounced in the lowest education group (*B* = .396, *p* = .018). For Social Development, significant interaction effects were found among parents with diploma-level education, both for TSS F1 (*B* = .385, *p* = .028) and TSS F2 (*B* = .372, *p* = .027), suggesting that school readiness perceptions among diploma holders were more strongly associated with social development outcomes than among those with a bachelor's degree or higher. No other significant interactions were found between parental education and CRSS subscales.

Regarding mental health, the effect of General Preparedness was significantly moderated by parent education level, with diploma-level parents showing a weaker relationship (*B* = 2.865, *p* = .037) (Table 5). This suggests that the positive impact of General Preparedness on mitigating children's mental health symptoms was less pronounced among parents with diploma-level education. All other interaction effects between school readiness and parental education level were non-significant.

**Table 5:** Significant interaction effects between predictors and sociodemographic factors in regression models.

Predictors	*B*	*SE B*	*β*	*t*	*p*	Partial *η*^2^
Parent’s age						
Predicting A/D-CBCL						
Constant	0.044	0.264	—	0.166	0.869	0.000
CRSS Social Development	−2.488	0.486	−0.317	−5.124	<0.001***	0.101
Age	−0.060	0.047	−0.080	−1.271	0.205	0.007
CRSS Social Development × Age	0.152	0.075	0.128	2.039	0.043*	0.018
Parent’s gender (female as reference group)						
Predicting A/D-CBCL						
Constant	−0.033	0.279	—	−0.119	0.905	0.000
CRSS General Preparedness	−2.459	0.497	−0.337	−4.945	<0.001***	0.087
Gender: male	0.332	0.711	0.030	0.467	0.641	0.001
CRSS General Preparedness × Gender: male	2.718	1.122	0.172	2.423	0.016*	0.023
Family income (high-income as reference group)						
Predicting CRSS General Preparedness						
Constant	0.068	0.064	—	1.057	0.291	0.004
TSS F1	0.220	0.113	0.198	1.942	0.053	0.015
Low-income	−0.035	0.085	−0.029	−0.410	0.682	0.001
Middle-income	−0.100	0.086	−0.083	−1.159	0.248	0.005
TSS F1 × Low-income	0.335	0.167	0.160	2.006	0.046*	0.016
TSS F1 × Middle-income	0.148	0.155	0.082	0.952	0.342	0.004
Parent’s education level (bachelor or above as reference group)						
Predicting CRSS General Preparedness						
Constant	0.014	0.070	—	0.200	0.842	0.000
TSS F1	0.210	0.121	0.189	1.739	0.083	0.012
Secondary education or below	0.006	0.083	0.005	0.074	0.941	0.000
Diploma	−0.010	0.102	−0.007	−0.096	0.923	0.000
TSS F1 × Secondary education or below	0.308	0.152	0.195	2.030	0.043*	0.016
TSS F1 × Diploma	0.028	0.183	0.012	0.151	0.880	0.000
Predicting CRSS Social Development						
Constant	−0.060	0.066	—	−0.903	0.367	0.003
TSS F1	0.141	0.115	0.133	1.227	0.221	0.006
Secondary education or below	0.083	0.079	0.075	1.046	0.297	0.004
Diploma	0.115	0.097	0.084	1.182	0.238	0.005
TSS F1 × Secondary education or below	0.246	0.144	0.163	1.702	0.090	0.011
TSS F1 × Diploma	0.385	0.174	0.171	2.207	0.028*	0.019
Predicting CRSS Social Development						
Constant	−0.061	0.068	—	−0.904	0.367	0.003
TSS F2	0.117	0.112	0.116	1.045	0.297	0.004
Secondary education or below	0.082	0.081	0.074	1.015	0.311	0.004
Diploma	0.098	0.100	0.072	0.987	0.325	0.004
TSS F2 × Secondary education or below	0.129	0.142	0.088	0.905	0.366	0.003
TSS F2 × Diploma	0.372	0.167	0.179	2.219	0.027*	0.019
Predicting CRSS Emotional and Behavioral Development						
Constant	0.032	0.077	—	0.413	0.680	0.001
TSS F1	0.115	0.132	0.095	0.869	0.386	0.003
Secondary education or below	−0.082	0.091	−0.064	−0.898	0.370	0.003
Diploma	0.144	0.112	0.093	1.291	0.198	0.006
TSS F1 × Secondary education or below	0.396	0.166	0.229	2.381	0.018*	0.022
TSS F1 × Diploma	0.128	0.201	0.049	0.636	0.525	0.002
Predicting A/D-CBCL						
Constant	−0.330	0.524	—	−0.630	0.529	0.002
CRSS General Preparedness	−2.907	0.889	−0.397	−3.270	0.001**	0.040
Secondary education or below	0.426	0.623	0.050	0.683	0.495	0.002
Diploma	0.144	0.765	0.014	0.188	0.851	0.000
CRSS General Preparedness × Secondary education or below	0.863	1.058	0.090	0.815	0.416	0.003
CRSS General Preparedness×Diploma	2.865	1.367	0.165	2.095	0.037*	0.017

Significant interaction effects emerged between predictors and sociodemographic factors in the regression models. After correction for multiple comparisons, only the interaction between CRSS General Preparedness and parent gender in relation to children’s mental health outcomes remained statistically significant. Uncorrected analyses suggested possible moderation by income and education; however, these findings should be interpreted cautiously and require replication in larger, more diverse samples.

TSS F1, Transition Strategies Scale Factor 1 (Curriculum and Administration); TSS F2, Transition Strategies Scale Factor 2 (Home-School Collaboration); CRSS, Chinese Readiness for School Scale; A/D-CBCL, Anxious/Depressed subscale of Child Behavior Checklist.

*p < .05, **p < .01, ***p < .001.

### 3.8 Sensitivity analyses

To evaluate the stability of the main findings, sensitivity analyses were conducted using bootstrapping. The data was repeatedly resampled, and the analysis was re-run 1,000 times to assess the robustness of the observed moderation effect related to parental education. Overall, the interaction effects of parents with diploma-level education and school readiness or mental health remained largely consistent. However, for the specific association between TSS F1 and CRSS General Preparedness, the result did not remain statistically significant. This suggests that while most findings were stable, certain effects, particularly those related to general preparedness, should be interpreted with caution, as their robustness may be sample- or model-dependent.

After applying Bonferroni corrections for multiple comparisons in the interaction tests, only the interaction between CRSS General Preparedness and parent gender in relation to children's mental health outcomes remained statistically significant. However, uncorrected analyses revealed several potential moderation patterns involving income and education that warrant replication in larger samples. The use of multiple comparisons corrections reflects appropriate statistical rigor and underscores the need for cautious interpretation in exploratory moderation research.

**Figure 3:**
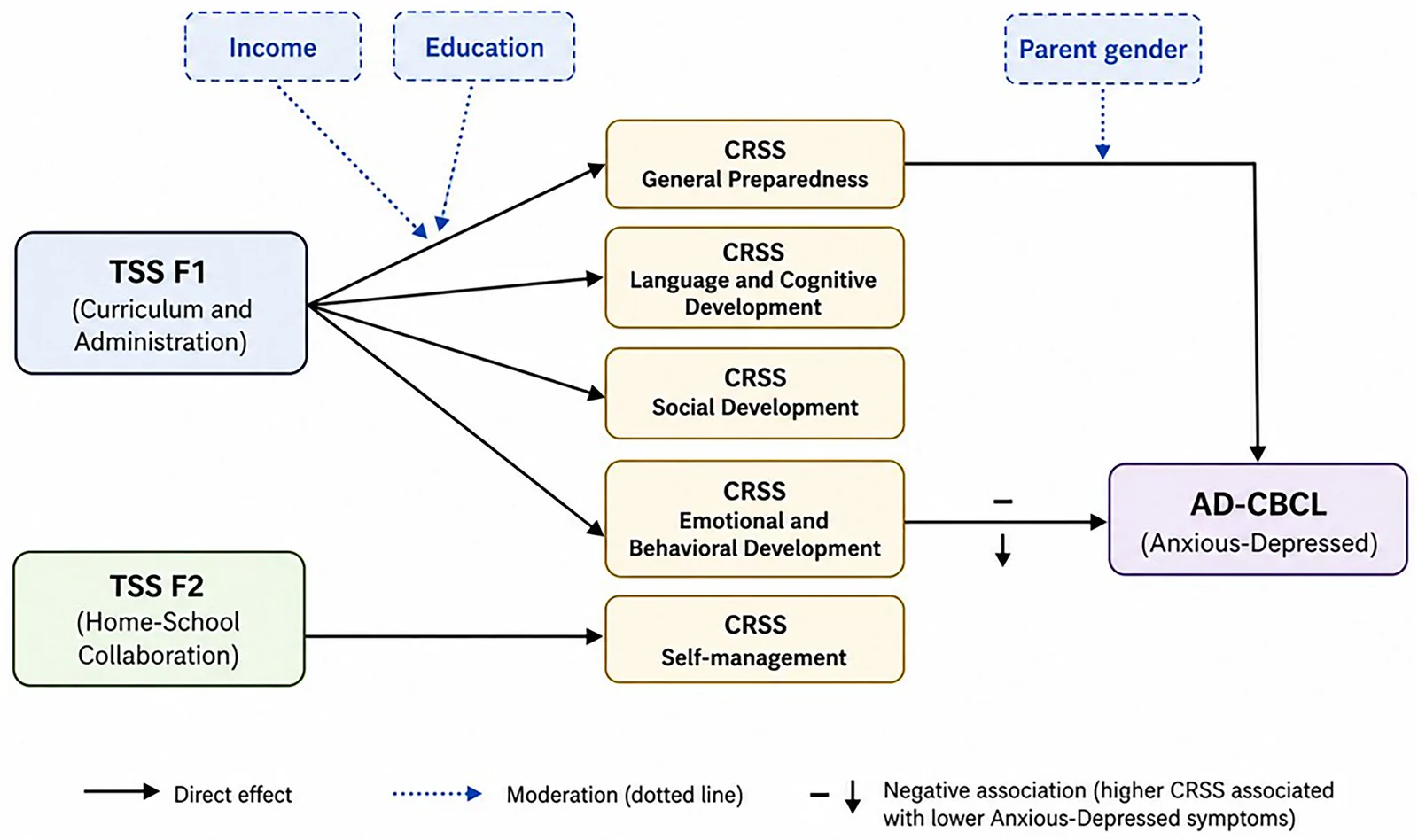
Overview of the study's key findings.

Figure 3 illustrates the key findings of the study.

## 4 Discussion

This study tested four hypotheses to examine the relationships among parental perceptions of transition practices, children's school readiness, and child mental health outcomes, as well as the moderating role of sociodemographic factors. H1 was supported: perceptions of transition practices related to curriculum and administration (TSS F1) were significantly associated with four domains of school readiness, while perceptions related to home–school collaboration (TSS F2) were uniquely associated with self-management. H2 was supported: higher parental perceptions of school readiness, particularly in socio-emotional domains, were associated with fewer child mental health difficulties. Emotional and Behavioral Development emerged as the strongest predictor, underscoring the protective role of socio-emotional readiness. H3 and H4 were partially supported: moderation analyses revealed that parent gender and education level influenced the strength of associations, with mothers and less educated parents perceiving school readiness factors differently in relation to child mental health outcomes. Household income also moderated the effect of TSS F1 on General Preparedness, with stronger associations observed among low-income families. Sensitivity analyses confirmed the robustness of most findings, although some effects (e.g., General Preparedness among diploma-level parents) were sample dependent, and only the gender moderation effect remained significant after correction for multiple comparisons. Taken together, these exploratory findings underscore the need to tailor transition practices to diverse family contexts and to ensure equitable support for children's adjustment. They suggest that socio-emotional readiness is a critical protective factor across groups, while differences by gender, education, and income point to the need for culturally and socioeconomically responsive interventions that promote equity in child development.

### 4.1 Transition practices and school readiness

The findings confirmed that parental perceptions of transition practices were shown to be closely linked to children’s school readiness. Curriculum and administration practices (TSS F1) were associated with multiple domains of school readiness, suggesting that structured, school-level supports play a broad role in fostering preparedness across cognitive, social, and emotional areas. In contrast, home–school collaboration (TSS F2) was uniquely linked to self-management, highlighting the importance of parental engagement and communication with schools in cultivating independence and self-regulatory skills among children (5). These findings confirm and extend prior research by demonstrating that different dimensions of transition practices contribute to distinct aspects of school readiness, underscoring the need for multifaceted transition strategies that integrate both school-level systems and family–school partnerships (5, 38).

### 4.2 School readiness and child mental health

Higher parental perceptions of school readiness were associated with better child mental health outcomes (60), with socio-emotional domains emerging as particularly protective. Emotional and Behavioral Development was the strongest predictor, with greater readiness in this area associated with fewer internalizing symptoms (61). This underscores the importance of emotional regulation, resilience, and adaptive behaviors in buffering children against anxiety and depression during the transition to primary school. The robustness of this effect across models highlights socio-emotional readiness as a critical foundation for adjustment and long-term wellbeing (62). Interventions that strengthen children's emotional and behavioral competencies may therefore be especially effective, pointing to the need for holistic transition practices that extend beyond academic preparation emphasized by Chinese parents to encompass socio-emotional development.

### 4.3 Gender differences in parental perceptions

Across CRSS subscales, mothers consistently reported higher school readiness scores than fathers, reflecting differences in caregiving roles and exposure to indicators of readiness. Fathers, who were predominantly employed full-time in this sample, may have had more limited direct involvement in the K-P transition process. In addition, fathers reported slightly higher levels of internalizing symptoms in children, as measured by the A/D-CBCL. This pattern may reflect gender-based perceptual differences in interpreting children's emotional expression or be influenced by paternal psychological distress, as prior research indicates that parental anxiety, depression, or elevated work-related stress can heighten sensitivity to children's emotional difficulties (63–65). These findings highlight the importance of considering gendered caregiving perspectives and parental well-being when interpreting school readiness and mental health outcomes, and underscore the need for future research to incorporate paternal voices more fully in transition studies.

### 4.4 Moderation by sociodemographic factors

Although H3 and H4 received only partial support, the moderation analyses provide important insights into how family context shapes the impact of parental perceptions of transition practices and school readiness on child mental health.

**Gender.** Mothers showed stronger protective associations between school readiness and reduced internalizing symptoms compared to fathers, consistent with prior work emphasizing gendered caregiving perspectives in interpreting children's preparedness and well-being (38).

**Education.** Parents with lower educational attainment reported stronger links between transition practices and school readiness, particularly in emotional and social domains. Families with fewer educational resources may therefore place greater value on structured transition practices to support their children's development, as low-SES households often have limited means to provide additional support. At the same time, the protective effect of General Preparedness on children's mental health appeared weaker among diploma-level parents. This pattern may reflect educational expectations and home–school interaction styles common among diploma-educated parents in Hong Kong, who often prioritize academic readiness over emotional readiness, which may lead them to perceive a weaker link between general preparedness and children's mental health. Sensitivity analyses using 1,000 bootstrap resamples indicated that most moderation effects involving parental education were stable; however, the specific association between TSS F1 and General Preparedness did not remain statistically significant, suggesting that this particular effect should be interpreted with caution. Overall, these findings highlight the role of parental education in shaping readiness perceptions and interpretations of their impact on children's well-being, consistent with prior evidence linking parental education levels to children's mental health outcomes (66).

**Income**. Household income moderated the effect of TSS F1 on General Preparedness, with stronger positive associations observed among low-income families. This suggests that curriculum- and administration-focused transition practices may play a particularly important role in supporting preparedness in households with fewer financial resources. Importantly, no moderation effects were found for other CRSS subscales or for the link between school readiness and child mental health, indicating that the influence of income is domain-specific. These findings highlight the potential of targeted transition practices to help mitigate socioeconomic disparities in school readiness, consistent with prior research showing that structured transition supports can buffer the disadvantages faced by children from economically constrained families (5, 38).

**Age**. Our results showed a significant interaction between CRSS Social Development and parent age. Specifically, higher levels of CRSS Social Development were strongly protective against depressive and anxiety symptoms among younger parents, but this protective effect was weaker among older parents. This pattern aligns with evidence that parental age shapes perceptions of children's socio-emotional and behavioral difficulties, with older fathers reporting lower distress but differing in their evaluations compared to younger fathers (67). Such differences in appraisal may help explain why the protective influence of social development weakens with age in our sample. One possible explanation is that older parents, drawing on greater life experience, may judge social development as less effective in mitigating internalizing symptoms, or may place greater emphasis on academic and behavioral competencies over social readiness. Generational differences in caregiving expectations could therefore account for the diminishing protective effect of social development with age. These findings underscore the importance of considering parental age when interpreting readiness perceptions, as developmental domains may be valued differently across age groups. Tailoring transition strategies to reflect these generational perspectives could enhance their relevance and effectiveness in promoting child well-being. Future studies should examine these age-related differences further, ideally with larger and more diverse samples.

Taken together, these moderation effects highlight the contextual nature of parental perceptions, consistent with ecological perspectives on child development. While robustness checks suggest caution in interpreting some associations, the observed patterns point to important directions for practice and research. Transition strategies that are responsive to socioeconomic disparities, educational differences, and caregiving perspectives may be particularly effective in promoting equitable readiness outcomes. Future studies should replicate these moderation effects in larger and more diverse samples and explore how culturally tailored interventions can strengthen both academic and socio-emotional readiness across family contexts.

### 4.5 Limitations and future directions

Several limitations should be considered when interpreting the findings of this study. First, the predominance of maternal participants may limit the generalizability of the results. Although this gender distribution reflects the reality of high maternal involvement in caregiving and aligns with patterns commonly observed in most existing research (68, 69), the relatively small sample size and the underrepresentation of fathers restrict the inclusion of paternal perspectives. This imbalance limits the representativeness of the findings and constrains our ability to capture potential differences between mothers' and fathers' perceptions of transition practices and school readiness, as well as their implications for children's mental health. Future studies should aim to recruit more balanced and diverse samples, with greater paternal participation, to enhance external validity and provide a more comprehensive understanding of parental perceptions.

Second, although schools were randomly selected from the Education Bureau's official list, parent participation within those schools was voluntary. This two-stage process reduced systematic bias at the school level but introduces potential bias at the parent level, thereby limiting the representativeness and generalizability of the findings. In addition, the response rate was not recorded during data collection, which constrains the ability to assess potential non-response bias. Only Chinese parents residing in Hong Kong were included, further limiting external validity. Future studies should employ stratified random sampling to ensure subgroup representativeness and replicate findings across diverse cultural contexts and other Chinese communities, such as those in mainland China.

Third, although the overall project was designed as a longitudinal mixed-methods study, the present manuscript reports findings from the first wave of cross-sectional data due to attrition in the subsequent wave. Attrition is a common challenge in longitudinal research, and in this case it restricted our ability to establish temporal ordering among parental perceptions of transition practices, children's school readiness, and children's mental health. As a result, the present analyses cannot support causal inference, and we interpret the observed associations as correlational rather than directional. Even so, the first-wave dataset remains robust and informative. In addition, the three-month interval between data collection points was relatively short, which would have limited our ability to capture meaningful developmental changes even with full retention. Future analyses incorporating additional waves of data over extended timeframes will be essential for strengthening internal validity, testing directional pathways, and examining developmental trajectories more comprehensively.

Fourth, the exclusive use of parent-reported measures may introduce potential bias due to subjectivity inherent in parents' ratings of transition practices, children's school readiness, and child mental health outcomes. Relying on a single informant also raises the possibility of shared-method variance, and parents' own psychological distress or expectations may have influenced their reports. Parental distress can lead to overestimation of children’s emotional difficulties, and the present study did not include measures of parents' mental health that could help account for such bias. These limitations constrain the objectivity and reliability of the findings. To enhance validity, future research should adopt multi-informant and multi-method designs that incorporate teacher evaluations, child self-reports, observational data, and assessments of parental mental health to triangulate parent-reported information and reduce subjective bias.

Fifth, although the A/D-CBCL instrument employed in this study is well-established and psychometrically robust, it may not capture the full spectrum of children's emotional health. Future research incorporating teacher assessments, clinician reports, or direct observational data could offer more triangulated and comprehensive insights into children's developmental functioning. It is also recommended that future studies employ the full CRSS, including the motor skills subscale, to more fully explore the relationship between motor development and school readiness.

Next, although household income tiers were classified using 2017 Census benchmarks to align with the data collection period, we acknowledge that income distributions may have shifted since then. Nevertheless, the classification remains meaningful for interpreting parental perspectives within the socioeconomic context of the study.

Finally, the study investigated a relatively limited set of variables. Future research would benefit from including additional mediators and moderators, such as parenting styles, parental beliefs about school readiness, and home-school involvement to deepen understanding of the multifaceted processes underlying school transition and emotional well-being.

Looking ahead, longitudinal studies that follow children's developmental trajectories over time are recommended to better understand the long-term impact of transition practices and parental perceptions. Expanding the sample size, ensuring broader demographic representation, particularly with respect to parental gender, and adopting multi-informant designs will enhance the robustness and generalizability of future findings. Further investigation into the mechanisms underlying the observed moderation effects by gender, education level, and household income may inform the development of more targeted, culturally responsive, and equitable interventions. Ultimately, such efforts could strengthen children's emotional and behavioral readiness, especially among vulnerable populations, and contribute to more inclusive early childhood education systems.

### 4.6 Implications for policy and practice

Although moderation effects by household income did not remain statistically significant after correction, preliminary patterns suggest that economically disadvantaged families may benefit more from structured transition practices. Policymakers should prioritize comprehensive, culturally responsive strategies that address both academic and socio-emotional readiness. Practical measures could include subsidized transition workshops, home–school liaison staff to strengthen family engagement, and take-home learning kits or digital resources to reinforce self-management and emotional development. Universal screening for emotional and behavioral challenges in the final year of kindergarten or early primary school would enable early identification and referral to counselling or community mental health services. Embedding parent-education components into transition planning may further reduce disparities in developmental outcomes. Finally, cross-sector collaboration among schools, health services, and NGOs is essential to ensure sustained support for children from disadvantaged backgrounds. Such measures can help promote equitable school readiness and mental health outcomes, ensuring that all children enter primary school with the socio-emotional competence and foundational skills needed for success.

## 5 Conclusion

This study examined the relationships among parental perceptions of transition practices, children's school readiness, and mental health, with attention to sociodemographic moderators. Findings provide important insights into the multifaceted nature of early educational transitions. Emotional and Behavioral Development consistently emerged as the strongest predictor of child mental health, underscoring the protective role of socio-emotional competence during the kindergarten–primary transition. Moderation analyses offered partial support for H3 and H4, revealing contextual influences of parental gender, education, and household income, although only the interaction between General Preparedness and parent gender remained statistically significant after Bonferroni correction. Sensitivity analyses further indicated that some associations may be sample- or model-dependent, highlighting the need for replication in larger and more diverse cohorts.

Overall, the findings suggest that socio-emotional readiness is a critical determinant of children's mental health during school transition, while the perceived effectiveness of transition practices varies across family contexts. Tailoring transition strategies to account for gendered caregiving perspectives and socioeconomic diversity may strengthen their impact and promote more equitable developmental outcomes.

## Data Availability

The original contributions presented in the study are included in the article/Supplementary Material, further inquiries can be directed to the corresponding author.
